# Altered nicotine reward-associated behavior following α4 nAChR subunit deletion in ventral midbrain

**DOI:** 10.1371/journal.pone.0182142

**Published:** 2017-07-31

**Authors:** Can Peng, Staci E. Engle, Yijin Yan, Marcus M. Weera, Jennifer N. Berry, Matthew C. Arvin, Guiqing Zhao, J. Michael McIntosh, Julia A. Chester, Ryan M. Drenan

**Affiliations:** 1 Department of Pharmacology, Northwestern University Feinberg School of Medicine, Chicago, Illinois, United States of America; 2 Department of Medicinal Chemistry and Molecular Pharmacology, Purdue University, West Lafayette, Indiana, United States of America; 3 Department of Psychological Sciences, Purdue University, West Lafayette, Indiana, United States of America; 4 George E. Wahlen Veterans Affairs Medical Center and Departments of Psychiatry and Biology, University of Utah, Salt Lake City, Utah, United States of America; Weizmann Institute of Science, ISRAEL

## Abstract

Nicotinic acetylcholine receptors containing α4 subunits (α4β2* nAChRs) are critical for nicotinic cholinergic transmission and the addictive action of nicotine. To identify specific activities of these receptors in the adult mouse brain, we coupled targeted deletion of α4 nAChR subunits with behavioral and and electrophysiological measures of nicotine sensitivity. A viral-mediated Cre/lox approach allowed us to delete α4 from ventral midbrain (vMB) neurons. We used two behavioral assays commonly used to assess the motivational effects of drugs of abuse: home-cage oral self-administration, and place conditioning. Mice lacking α4 subunits in vMB consumed significantly more nicotine at the highest offered nicotine concentration (200 μg/mL) compared to control mice. Deletion of α4 subunits in vMB blocked nicotine-induced conditioned place preference (CPP) without affecting locomotor activity. Acetylcholine-evoked currents as well as nicotine-mediated increases in synaptic potentiation were reduced in mice lacking α4 in vMB. Immunostaining verified that α4 subunits were deleted from both dopamine and non-dopamine neurons in the ventral tegmental area (VTA). These results reveal that attenuation of α4* nAChR function in reward-related brain circuitry of adult animals may increase nicotine intake by enhancing the rewarding effects and/or reducing the aversive effects of nicotine.

## Introduction

Nicotinic acetylcholine receptors (nAChRs) participate in cholinergic modulation of neuronal activity and mediate the response to nicotine. In neurons of the midbrain dopamine (DA) system, they are found in postsynaptic and presynaptic locations, and three main heteromeric nAChR subtypes exist (* indicates the possibility of other subunits besides those that are named): α4β2(non-α6)*, α6β2(non-α4)*, and α4α6β2*. Activation of α4β2* nAChRs is both necessary and sufficient for nicotine reward in mice [1–3]. Because the biophysical and pharmacological features of α4β2* and α6β2* nAChR subtypes differ [4, 5], and because drugs are actively being sought to activate/modulate these receptors for human disorders/diseases [6], it is important to delineate their functional roles in the DA system.

Previous studies point to differential expression patterns and functional roles for α4β2(non-α6)*, α6β2(non-α4)*, and α4α6β2* nAChR subtypes. For example, α4 nAChR subunits appear to be expressed in nearly all ventral tegmental area (VTA) and substantia nigra neurons [7], whereas α6 nAChR subunits appear to be more preferentially expressed in DA (but not GABA) neurons [8, 9]. Exposure of α4β2(non-α6)* nAChRs to nicotine results in desensitization at lower nicotine concentrations than are required to activate the receptor [10]. In contrast, incorporation of α6 subunits into α4β2* nAChRs may confer resistance to desensitization [11]. α4β2* are known to up-regulate in response to chronic nicotine treatment, while α6β2* nAChRs down-regulate following such treatment [4]. Studies utilizing mice with hypersensitive α4 or α6 subunits reveal that selective activation of α4β2* versus α6β2* nAChRs causes different behavioral phenotypes. For example, selective activation of α4β2* nAChRs causes locomotor suppression [1], whereas selective activation of α6β2* nAChRs robustly stimulates locomotor activity [9, 12–14]. Together, these results suggest that these nAChR subtypes are expressed in different VTA neurons, and contribute to diverse functions and/or activities. Uncovering these activities could lead to novel approaches to treat disorders involving DA system nAChRs.

Functional roles for native nAChR subtypes are most commonly inferred from loss-of-function studies involving pharmacological antagonism and/or gene knockout (KO) techniques. Approaches involving the latter usually rely on KO mice that have had the nAChR subunit under investigation knocked out at the single cell stage and throughout all of development. This can result in developmental adaptations in protein expression/function, confounding data analysis and interpretation. Other approaches that isolate specific nAChRs in vivo, such as the use of mice expressing hypersensitive nAChRs [15], also have the potential confound that normal physiological activity of the target receptor is altered. To overcome these limitations, we studied mice homozygous for a “floxed” version of the α4 nAChR subunit gene [16]. By creating mice with conditional deletion of α4* nAChRs in specific brain areas after mice reach adulthood, and by studying them shortly after deletion, we bypassed potential developmental artifacts associated with traditional KO mice. We used this approach to study nicotine’s behavioral motivational effects following α4 conditional deletion in the ventral midbrain, and electrophysiological experiments were used to identify potential mechanisms associated with the identified behavioral phenotypes.

## Materials and methods

### Materials

(-) Nicotine hydrogen tartrate salt was obtained from Glentham Life Sciences (Wiltshire, United Kingdom) and (-) nicotine base was obtained from Acros Organics (Geel, Belgium). All nicotine doses are reported as free base doses. 6-cyano-7-nitroquinoxaline-2,3-dione (CNQX) was purchased from Tocris Biosciences (Ellisville, MO). α-Conotoxin MII (MII) was synthesized as previously described [17]. All other chemicals without a specified supplier were obtained from Sigma (St. Louis, MO).

### Mice

All experimental protocols involving mice were reviewed and approved by the Institutional Animal Care and Use Committee at Purdue University (protocol #1110000074) or Northwestern University (protocol #IS00003282). Procedures also followed the guidelines for the care and use of animals provided by the National Institutes of Health Office of Laboratory Animal Welfare. All efforts were made to minimize animal distress and suffering during experimental procedures, including during the use of anesthesia (ketamine, xylazine, pentobarbital). Mice were housed at 22°C on a 12-hour light/dark cycle with food and water *ad libitum*. Mice were weaned on postnatal day 21 and housed with same-sex littermates. A tail sample was taken from each mouse for genotyping via polymerase chain reaction (PCR) as previously described [9, 16]. Mice homozygous for floxed α4 alleles, studied previously [16], are referred to hereafter as α4-flox mice. In these mice, loxP sites flank exon 5 of the *Chrna4* gene, allowing for conditional deletion of α4 in germline or somatic cells that express Cre recombinase. In this study, α4 nAChR subunits were eliminated in neurons of α4-flox mice by injecting viral vectors driving Cre recombinase expression into a brain region of interest. C57BL/6 wild type mice were purchased from Jackson Laboratories and were used to establish doses for nicotine conditioned place preference.

### Stereotaxic surgery

Male and female mice were used for surgery starting at 8 weeks of age. Mice were initially anesthetized with an intraperitoneal (i.p.) injection of a ketamine/xylazine mixture (120 mg/kg ketamine, 16 mg/kg xylazine). Mice were given additional “boost” injections of ketamine (100 mg/kg, i.p.) as needed. Mice were secured into a stereotaxic frame and a small incision at the top of the head was made to expose the skull. Coordinates used for VTA injections were: M/L: ±0.5 mm from bregma, A/P: -3.2 mm from bregma, D/V: -4.5 mm from bregma. Exact coordinates were adjusted to account for slight differences in the head size of individual mice: the bregma/lambda distance measured for each mouse was divided by the reported bregma/lambda distance for C57 mice (4.21), then multiplied by the A/P coordinate. The injection needle was slowly lowered through the drilled hole to the D/V coordinate. For the AAV-GFP and AAV-Cre-GFP viruses (AAV2.CMV.PI.eGFP.WPRE.bGH and AAV2.CMV.HI.eGFP-Cre.WPRE.SV40 purchased from Penn Vector Core), 250 nL of virus was infused at a rate of 50 nL/min. The injection needle was left in place for 5 min after the infusion ended before slowly retracting the needle. Sutures were used to close the incision. At the conclusion of the surgery, mice were given ketoprofen (5 mg/kg, s.c.) and placed in a recovery cage, kept warm, and observed until they were ambulatory. Mice were single-housed following virus injection surgery and were given at least 14 days to recover and for the virus to express before beginning experimental procedures. Accurate targeting of VTA with viral vectors was assessed in all mice. For electrophysiology experiments, accurate targeting of VTA was determined via direct visualization of fluorescent neurons in brain slices during recordings. Slices without such neurons were not used for recordings. For behavior experiments, accurate targeting of VTA was determined by anti-GFP immunohistochemistry.

### Oral nicotine self-administration

Two bottle choice nicotine drinking was conducted similar to a previous study [18]. Mice [24 α4-flox;Cre(+) (11 male/13 female) and 26 α4-flox;GFP(+) (13 male/13 female)] were single-housed for 14 days with free access to food and water before the start of drinking procedures to allow acclimation to the experimental environment and recovery from stereotaxic surgery. On day 15, the water bottle in each cage was replaced with two drinking tubes (25-mL graduated cylinders fitted with drinking spouts): one containing nicotine and the other containing water. The concentration of nicotine was increased every four days: days 1–4 (10 μg/mL), days 5–8 (25 μg/mL), days 9–12 (50 μg/mL), days 13–16 (100 μg/mL), days 17–20 (150 μg/mL), and days 21–24 (200 μg/mL). Intake volumes of nicotine and water were recorded daily. Every two days, mice were weighed, fluids were replaced, and drinking tube positions were swapped to prevent development of side preference. Non-consumption-related fluid loss was accounted for during analysis by placing two drinking tubes in an empty cage.

### Conditioned place preference (CPP)

Three-chamber place conditioning compartments were from Med Associates, Inc. (VT, USA). End chambers (16.764 cm x 12.7 cm x 12.7 cm) had either black walls with a grid rod style floor or white walls with a mesh style floor. The center chamber (7.24 cm x 12.7 cm x 12.7 cm) had a grey smooth acrylic plastic floor and manually retractable guillotine doors separated the center chamber from the end chambers. Photobeams spaced throughout the place conditioning compartment, coupled with MED-PC software, recorded the time spent and locomotor activity of a mouse in each chamber.

A biased place conditioning protocol was chosen based on a previous comparison between place conditioning using biased vs. unbiased procedures [19], as well as recent studies in the field of nAChR behavioral pharmacology that employed a biased procedure [1, 20–24]. CPP in C57Bl/6 WT mice was restricted to males (n = 36) to allow us to identify a dose of nicotine for subsequent place conditioning studies in α4-flox mice. CPP in α4-flox mice was conducted in mixed-sex groups as follows due low availability of homozygous α4-flox mice: AAV-GFP (n = 10; n = 5 male; n = 5 female), AAV-Cre-GFP (n = 10; n = 6 male; n = 4 female). All mice used for CPP were singly housed and restricted to 4 grams of food pellets per day. This amount of food maintained mice at ~90% normal body weight. In rat, nicotine intravenous self-administration is often accomplished associated with food restriction [25]. Food restriction has also been used in mouse place conditioning studies [26]. Mice were weighed throughout the study, and we designated 80% of starting weight as a cutoff such that any mouse dropping below this cutoff was to be removed from the study. No mouse met this criterion during our studies, so no mice were excluded based on body weight. Since single housing can be anxiogenic, a “cave” and a cotton nestlet were included in the cage. Prior to the pre-test, mice were subjected to daily handling for three days. On day 1 (pre-test), mice were placed in the center chamber with the guillotine doors retracted, and mice were allowed to freely explore all chambers of the compartment for 15 min. Animals were then assigned to be conditioned with nicotine in their less-preferred end chamber based on the initial preference shown in the pre-test. On days 2–4 (conditioning), animals were conditioned twice a day with an interval of at least 4 hours. For nicotine-conditioned groups, in the morning, animals were injected (s.c.) with nicotine or saline and immediately confined in their assigned end chamber for 20 min. In the afternoon, animals were alternatively injected with saline or nicotine and immediately confined in the opposite end chamber for 20 min. For saline-conditioned control group, animals received saline injections in both end chambers. On day 5 (post-test), mice were placed in the center chamber with the guillotine doors retracted, and were allowed to freely explore the compartment for 15 min. The pre-test and post-test took place at mid-day. The total time spent in each end chamber was measured, and the difference in time spent in the conditioning chamber between the post-test and the pre-test was calculated and expressed as the place preference score.

### Brain slice patch clamp electrophysiology

All mice to be used for brain slice electrophysiology were deeply anesthetized with sodium pentobarbital (200 mg/kg, i.p.) to allow extraction of the brain for slice preparation. Coronal VTA brain slices (200 μm), prepared as previously described [27], were cut with a vibratome (DTK-Zero1 or Leica VT1200S) 2–3 weeks after AAV infusions. Patch clamp electrophysiology was carried out as previously described [27, 28]. For recording ACh-evoked currents, the following internal solution was used (in mM): 135 K^+^ gluconate, 5 EGTA, 0.5 CaCl_2_, 2 MgCl_2_, 10 HEPES, 2 MgATP, and 0.1 GTP; pH adjusted to 7.25 with Tris base; osmolarity adjusted to 290 mOsm with sucrose. We recorded from VTA DA neurons in lateral VTA, which is known to contain ~95% DAergic neurons [29]. A Nikon Eclipse FN-1 upright microscope using infrared or visible differential interference contrast (DIC) optics was used to visualize cells. A mercury or light emitting diode (LED) light source coupled to an excitation filter for GFP was used to identify infected neurons. pCLAMP 10.3 software (Molecular Devices; Sunnyvale, CA) was used to acquire whole-cell recordings along with an Axopatch 200B or Multiclamp 700B amplifier and a 16-bit Digidata 1440 A/D converter (all from Molecular Devices). To record ligand-activated currents, ACh was locally applied by pressure ejection to recorded neurons, as previously described [27, 28]. Atropine (1 μM) was present in the superfusion medium when using ACh to prevent activation of muscarinic ACh receptors. There was no apparent difference between ACh-evoked response amplitudes from infected vs. neighboring uninfected VTA neurons in slices from AAV-GFP infected animals, so to minimize animal usage and suffering, we pooled ACh-evoked current responses from these two conditions.

### AMPA/NMDA ratio measurements

AMPA/NMDA recordings were done using an internal solution containing (in mM): 117 CsCH_3_SO_3_, 20 HEPES, 0.4 EGTA, 2.8 NaCl, 5 TEA-Cl, 2.5 MgATP, and 0.25 MgGTP; pH adjusted to 7.25 with Tris base; osmolarity adjusted to 290 mOsm with sucrose. AMPA to NMDA ratio recordings were carried out as previously described [30, 31]. After virus injection surgery and recovery, mice were habituated to handling for 5 d immediately prior to the experiment day. On days 1–3 of handling mice were picked up and held, on day 4 they were scruffed and given a mock injection, and on day 5 mice were scruffed and given a saline injection. On the day of the experiment mice were given an injection of vehicle (saline) or nicotine (i.p., 0.17 mg/kg). Sixty min after the injection, mice were deeply anesthetized with sodium pentobarbital (200 mg/kg, i.p.) and the brain was dissected to prepare coronal VTA brain slices. A concentric bipolar stimulating electrode (FHC cat. #CBAPC75) was placed between 100–200 μm lateral to the VTA (in or near the medial lemniscus). Whole-cell recordings were established from VTA DA neurons. EPSCs were stimulated (250–750 μA, 0.1 Hz, 40 μsec) at a strength that produced currents at half their maximal amplitude. EPSCs were evoked at -70 mV and +40 mV. Neurons were initially held at a command voltage of -70 mV and 5 EPSCs were evoked. The neuron was then depolarized to +40 mV. After the neuron stabilized, 5 EPSCs were evoked at +40 mV. The 5 responses were averaged and used to determine the AMPA/NMDA ratio. Due to Mg^2+^ block of NMDARs at hyperpolarized command potentials, the responses at -70 mV should be entirely mediated by AMPARs. For that reason, the time of the peak at -70 mV was noted and the amplitude on the +40 mV trace at this time point is the AMPA component of the ratio. The +40 mV EPSCs are a combination of AMPAR and NMDAR-mediated responses. The NMDA component of the ratio was calculated as the average of the amplitude over a 10 msec window, 40 msec after the peak. By this time, the AMPAR-mediated EPSCs recorded at -70 mV had decayed backed to baseline so the remaining current at +40 mV should reflect only the NMDAR-mediated component. Picrotoxin was present in the superfusion medium to block inhibitory inputs.

### Immunohistochemistry and confocal microscopy

Mice were anesthetized with sodium pentobarbital (200 mg/kg, i.p.) and transcardially perfused with 10 mL of heparin-containing phosphate buffered saline (PBS) followed by 30 mL of 4% paraformaldehyde. Brains were dissected and postfixed in 4% paraformaldehyde overnight at 4°C. Coronal brain slices (50 μm) were cut on a vibratome (DTK-Zero1; Ted Pella). VTA-containing slices were stained using the following procedure. Slices were first permeabilized for 2 min via incubation in PBST (0.3% Triton X-100 in PBS), followed by a 60 min incubation in blocking solution (0.1% Triton X-100, 5% horse serum in Tris-buffered saline (TBS)). Primary antibodies used in this study were as follows: sheep anti-TH (Millipore AB1542), rabbit anti-GFP (Invitrogen A11122), rabbit anti-GAD65/67 (Sigma-Aldrich G5163). Primary antibodies were diluted in blocking solution (anti-TH at 1:800, anti-GFP at 1:500, anti-GAD65/67 at 1:1000). Slices were incubated in primary antibodies overnight at 4°C. Three 5 min washes in TBST (0.1% Triton X-100 in TBS) were done before transferring slices to secondary antibodies for a 60 min incubation at room temperature (anti-sheep or anti-rabbit Alexa 555, anti-rabbit Alexa 488, diluted to 1:500 in blocking solution). Slices were washed as before, mounted on slides, and coverslipped with Vectashield. Staining in the VTA was imaged as previously described [32] with a Nikon A1 laser-scanning confocal microscope.

To verify correct targeting of VTA with viral vectors, the above procedure was modified as follows. Mice were perfused as described above, brains were removed and placed in 4% paraformaldehyde overnight at 4°C. Brains were then placed into 30% sucrose at 4°C to dehydrate brains, which was confirmed when the brain sank to the bottom of the container. Brain slices (50 μm) were cut on a freezing microtome. Slices were washed three times in PBS for 10 min followed by 30 min incubation in 0.3% H_2_O_2_ in PBS. Following another three 10 min washes in PBS, slices were incubated in permeabilization buffer (20 mM HEPES, 50 mM NaCl, 3 mM MgCl_2_, 300 mM sucrose, 0.5% Triton X-100, pH 7.4) for 60 min at 4°C. Slices were then placed in a blocking solution (5% horse serum in TBST) for 30 min at 4°C followed by incubation in primary antibody (rabbit anti-GFP 1:500) diluted in blocking solution overnight at 4°C. The next day, slices were washed three times in TBST for 10 min followed by incubation in secondary antibody (biotinylated anti-rabbit 1:500) for 60 min at room temperature. Following another three 10 min washes in TBST, slices were incubated in Vectastain ABC reagent (Vector Laboratories, product number PK-6100; Burlingame, CA) for 30 min. After a final set of three 10 min washes in TBST, slices were placed in DAB substrate kit (Vector laboratories, product number SK-4100) for approximately 2 min. Slices were placed in distilled water for at least 5 min, mounted onto slides, and coverslipped using VectaMount. Visualization of staining and verification of virus placement was accomplished via a dissecting microscope.

### Single-cell reverse transcription PCR

Individual lateral VTA neurons were aspirated into a pipette containing the K^+^ gluconate-based internal solution prepared with diethylpyrocarbonate-treated water. The cell was expelled into ice-cold 75% ethanol, and RNA was precipitated at -20°C for 3 hours followed by centrifugation at 7500 g for 10 min at 4°C. First-strand cDNA was synthesized via reverse transcription (RT) (Sensicript RT Kit, Qiagen, Germantown, MD) using the oligo(dT)15 primer. A nested PCR strategy was used to amplify glutamic acid decarboxylase (GAD65), tyrosine hydroxylase (TH), and glyceraldehyde-3-phosphate dehydrogenase (GAPDH). In the first round of nested PCR, the single-cell cDNA was amplified (1 cycle: 94°C for 2 min; 20 cycles: 94°C for 30 s, 56°C for 30 s, and 72°C for 30 s; 72°C for 10 min) with the following primers: GAD65_F (TCT TTT CTC CTG GTG GCG CC), GAD65_R2 (CCC CAA GCA GCA TCC ACG T), TH_F (CAG TGA TGC CAA GGA CAA GC), TH_R2 (GAG AAG GGG CTG GGA ACT TT), GAPDH_F2 (AAC TTT GGC ATT GTG GAA GG), and GAPDH_R2 (CCC TGT TGC TGT AGC CGT AT). The GAD65, TH and GAPDH signals were further amplified in the second round of nested PCR (1 cycle: 94°C for 2 min; 36 cycles: 94°C for 30 s, 61°C for 30 s, and 72°C for 30 s; 72°C for 10 min) with the following primers: GAD65_F (TCT TTT CTC CTG GTG GCG CC), GAD65_R1 (GCA GCT CCC TTC TTG AGA GA), TH_F (CAG TGA TGC CAA GGA CAA GC), TH_R1 (CCT GTG GGT GGT ACC CTA TG), GAPDH_F1 (GTG TTC CTA CCC CCA ATG TG), and GAPDH_R1 (GGT CCT CAG TGT AGC CCA AG). Final PCR products were detected by 2% agarose gel electrophoresis.

### Statistics and data analysis

α level was set at 0.05 for all statistical tests, which were done using GraphPad Prism 7 (La Jolla, CA) software or IBM SPSS 24 (North Castle, NY). Statistical tests included unpaired student’s *t*-test, paired *t*-test, analysis of variance (ANOVA) and, where appropriate, post-hoc testing. All error bars denote SEM. Supporting Information files include behavioral data shown (S2, S3 and S4 Figs) separated by sex. Outlier analysis for oral nicotine self-administration was conducted as previously described [33, 34]. Electrophysiology traces were initially viewed using Clampfit (Molecular Devices) and were subsequently imported into, and plotted using, Origin 2017 (Northampton, MA) software.

## Results

**Fig 1:**
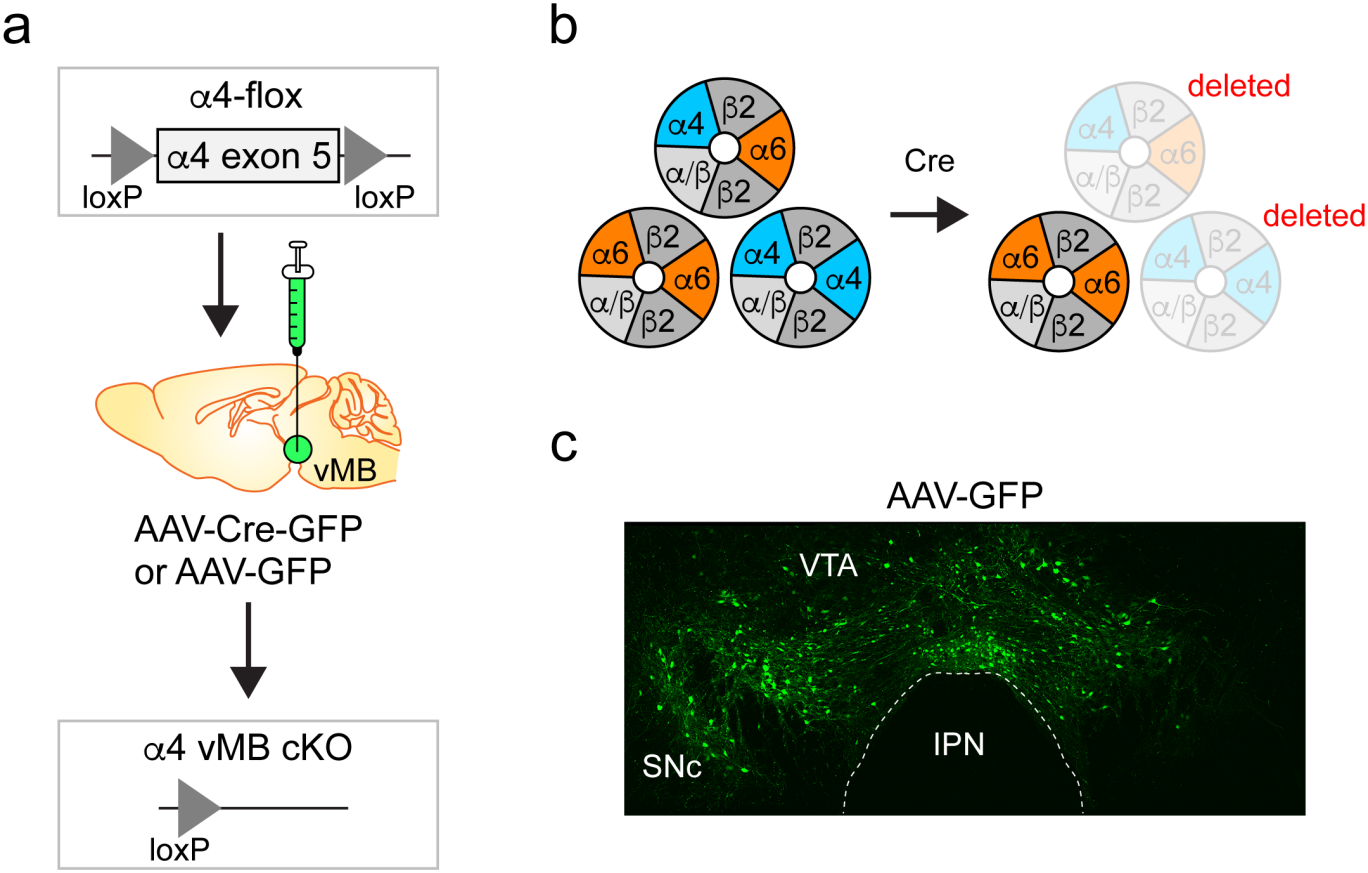
Conditional KO of α4 nAChR subunits. a) α4-flox mice have loxP sites flanking exon 5 of the α4 nAChR gene. Infusion of viral vectors expressing Cre recombinase into the VTA results in conditional deletion of α4 in infected cells. b) The three main heteromeric nAChR subtypes found in neurons of the midbrain DA system, α4α6β2*, α6 β2(non-α4)*, and α4β2(non-α6)*, are shown. Deletion of α4 eliminates α4α6β2* and α4β2(non-α6)* subtypes, while isolating the activity of α6β2(non-α4)* nAChRs. c) Viral spread of AAV-GFP vectors. An AAV-GFP virus was infused bilaterally into the VTA of a C57BL/6 WT mouse, followed 3 weeks later by perfusion and anti-GFP staining of brain sections.

In these studies, adeno-associated virus (AAV) constructs were used to express a Cre recombinase variant fused in-frame to GFP (Cre-GFP) in the ventral midbrain (vMB) of α4-flox mice, deleting α4* nAChRs in infected neurons (Fig 1a and 1b). Cre-GFP is referred to hereafter as “Cre”. Although we targeted the VTA, there was modest viral spread into the substantia nigra (Fig 1c). Therefore, we refer to the conditional deletion of α4 as encompassing the “ventral midbrain”.

**Fig 2:**
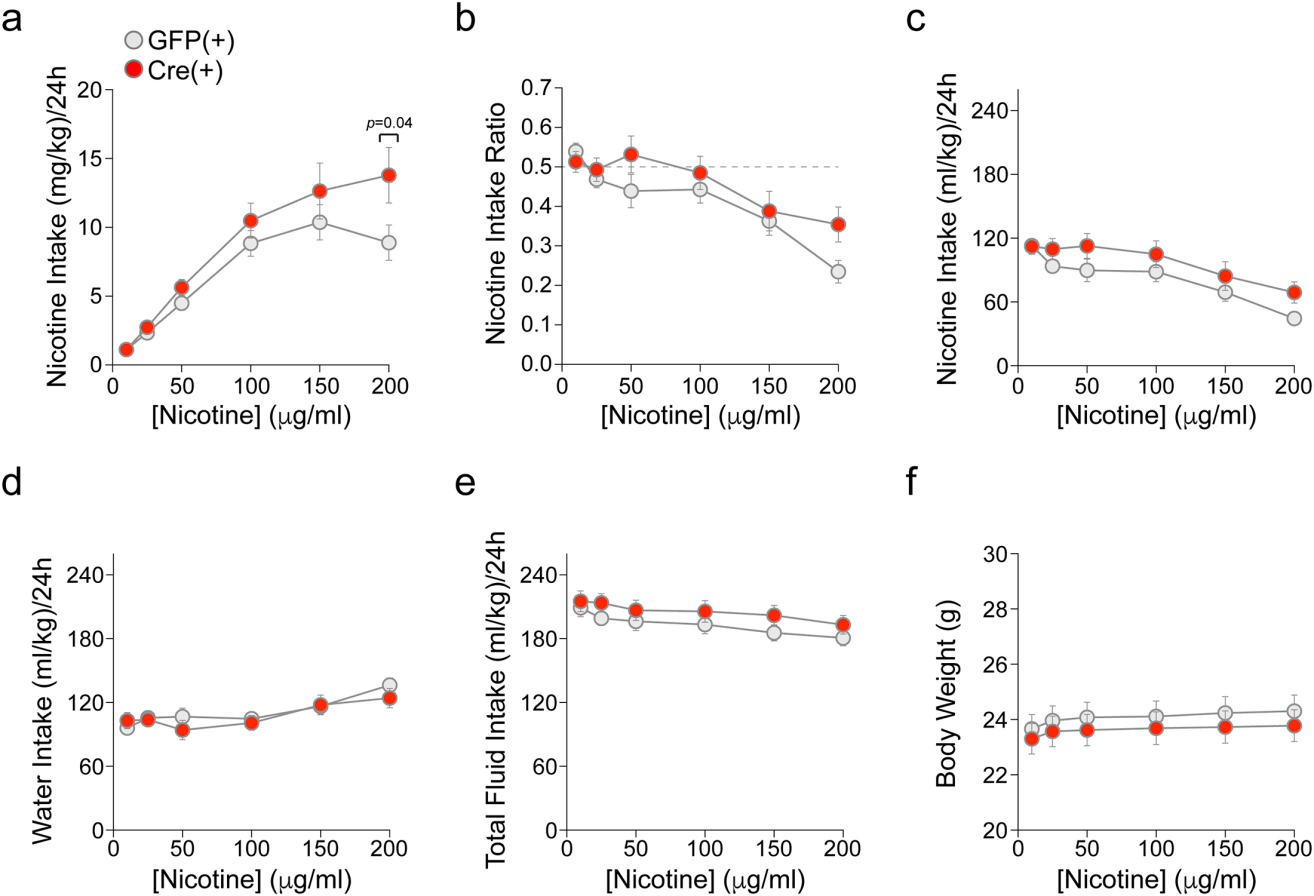
Enhanced nicotine oral SA in mice with vMB α4 deletion. a) Mean (± SEM) nicotine intake (mg/kg) over 4 days is shown for each nicotine concentration, in male and female α4-flox GFP(+) (n = 26) and Cre(+) (n = 24) mice. *p<0.05. b) Mean (± SEM) nicotine intake ratio (fraction of nicotine-containing fluid consumed relative to total fluid consumed) over 4 days is shown for each nicotine concentration in α4-flox GFP(+) and Cre(+) mice. c) Mean (± SEM) intake of nicotine solution (mL/kg), used to calculate mass of nicotine intake shown in (a), is shown for each nicotine concentration in α4-flox GFP(+) and Cre(+) mice. d) Mean (± SEM) water intake (mL/kg) over 4 days is shown for each nicotine concentration in α4-flox GFP(+) and Cre(+) mice. e) Mean (± SEM) total fluid intake (mL/kg) over 4 days is shown for each nicotine concentration in GFP(+) and Cre(+) mice. f) Mean (± SEM) body weight (g) for α4-flox GFP(+) and Cre(+) mice is shown for each nicotine concentration. S2, S3 and S4 Figs show intake data separated by sex.

To study the behavioral effect of this manipulation, we studied oral nicotine self-administration (SA) in α4-flox mice following conditional deletion of α4. α4-flox;GFP(+) and α4-flox;Cre(+) mice were habituated to the testing room and subsequently subjected to a continuous-access 2-bottle choice nicotine-drinking assay. α4-flox;GFP(+) mice showed a pattern of oral nicotine consumption (Fig 2a) consistent with previous studies that examined C57BL/6 mice [18]. Interestingly, α4-flox;Cre(+) mice consumed significantly more nicotine compared to their GFP(+) counterparts at the 200 μg/mL nicotine concentration (Fig 2a). ANOVA of nicotine concentration (6) x genotype (2) x sex (2) showed significant main effects of concentration [*F*(5,230) = 60.6, *p*<0.001] and sex [*F*(1,46) = 5.4, *p* = 0.02; females > males] and significant concentration x genotype [*F*(5,230) = 2.2, *p* = 0.05] and concentration x sex [*F*(5,230) = 2.7, *p* = 0.02] interactions. Follow up one-way ANOVAs of genotype within each concentration showed significantly greater intake of nicotine (mg/kg) in the α4-flox;Cre(+) mice vs. the GFP(+) mice at the 200 μg/mL concentration [*F*(1,49) = 4.3, *p* = 0.04] (Fig 2a). One-way ANOVAs of sex at each concentration revealed that the concentration x sex interaction was due to significantly greater intake of nicotine in females than males at each concentration except 200 μg/mL [*F*s(1,49)>4.1, *p*s<0.05].

Nicotine intake ratios were initially ~50% in both genotypes and gradually declined with increasing nicotine concentrations (Fig 2b). ANOVA of nicotine concentration (6) x genotype (2) x sex (2) only yielded a significant main effect of concentration [*F*(5,230) = 19.9, *p*<0.001], although the intake ratio was higher in the α4-flox;Cre(+) mice vs. the GFP(+) mice at the 200 μg/mL concentration (Fig 2b). Three-way ANOVAs of mL/kg intake of nicotine solution (Fig 2c), water (Fig 2d), and total fluid (Fig 2e) yielded main effects of nicotine concentration [*F*s(5,230)>8.8, *p*s<0.001] and sex [*F*s(1,46)>7.7, *p*s<0.01; females > males], but no main effects or interactions with genotype. Body weight was also not significantly different between the two genotypes (Fig 2f). All oral SA results are included in S1 File.

**Fig 3:**
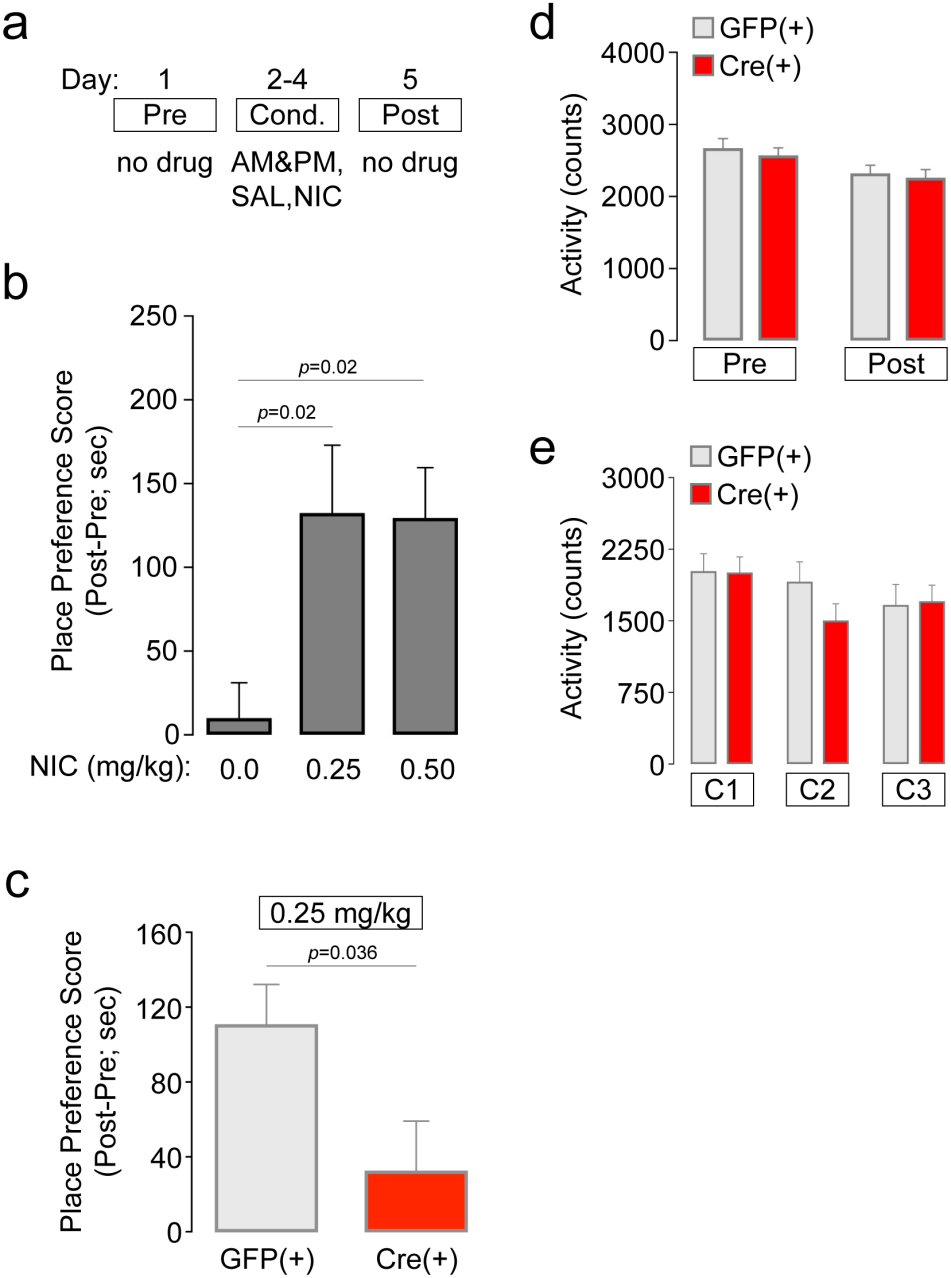
Nicotine CPP is reduced in mice with vMB α4 deletion. a) CPP schematic. Prior to a 5-day, biased CPP procedure to establish nicotine CPP, mice were mildly food-restricted and handled. Drug-free pre-test and post-test days flanked 3 consecutive conditioning days that consisted of morning and afternoon saline (SAL) and nicotine (NIC) conditioning sessions. b) Nicotine CPP in C57Bl/6 WT mice. Groups of WT mice were conditioned with saline (n = 12), 0.25 mg/kg NIC (n = 12), or 0.5 mg/kg NIC (n = 12) to validate our CPP assay and identify a dose to be used subsequently in α4-flox mice. Mean (± SEM) place preference score is shown for the three drug doses. *p* values are for Dunnett’s multiple comparisons test. c) Nicotine CPP in α4-flox mice. AAV-GFP or AAV-Cre-GFP vectors were infused into vMB of α4-flox mice (GFP(+), n = 10; Cre(+), n = 10), and mice were subsequently conditioned with 0.25 mg/kg NIC. Mean (± SEM) place preference score is shown for both groups. *p* value is for unpaired t-test. d) Mean (± SEM) locomotor activity during the pre-test and post-test is shown for α4-flox mice conditioned with NIC. e) Mean (± SEM) locomotor activity during conditioning sessions 1, 2, and 3 is shown for α4-flox mice conditioned with NIC.

Next, we complemented our studies of free-choice nicotine consumption (Fig 2) with nicotine place conditioning experiments. Place conditioning is advantageous for examining the motivational effects of abused drugs, including nicotine [35–38]. Here, we examined acquisition of nicotine CPP in mice using a 5 d, biased CPP design (Fig 3a). First, we established our nicotine CPP procedure in C57BL/6 WT mice. In male C57BL/6 WT mice, saline did not result in place conditioning, but nicotine (0.25 mg/kg and 0.5 mg/kg, s.c.) induced robust place conditioning (Fig 3b), as measured by place preference score (difference in time spent on the drug paired floor after conditioning minus before conditioning). ANOVA revealed a significant effect of treatment dose [*F*(2,33) = 4.889, *p* = 0.014], and Dunnett post-hoc testing indicated a significant increase in preference score in the 0.25 mg/kg (*p* = 0.02) and 0.5 mg/kg (*p* = 0.02) treatment groups relative to the control group. These data are consistent with previous work [1, 39]. From these results, we selected 0.25 mg/kg for further CPP testing in α4-flox mice. Groups of mixed sex α4-flox;GFP(+) and Cre(+) mice were conditioned with 0.25 mg/kg nicotine. α4-flox;GFP(+) mice acquired nicotine CPP (Fig 3c), as measured by difference score, in a manner very similar to C57BL/6 WT mice (Fig 3b). In contrast, difference scores were significantly less in α4-flox;Cre(+) mice, suggesting either reduced or no CPP (Fig 3c) (unpaired *t*-test: *p* = 0.036, *t* = 2.271). This analysis was likely not sufficiently powered to detect an effect within each sex, as genotype (2) x sex (2) two-way ANOVA did not detect a significant main effect of sex, genotype, or an interaction between the two. CPP results in α4-flox;GFP(+) and α4-flox;Cre(+) male and female mice are included in the Supporting Information (S5 Fig). There were no differences in locomotor activity between the GFP(+) and Cre(+) groups during any of the CPP phases (Fig 3d and 3e), suggesting that the CPP results are not the result of a locomotor deficit in either the GFP(+) or Cre(+) mice. All CPP results are included in S2 File.

**Fig 4:**
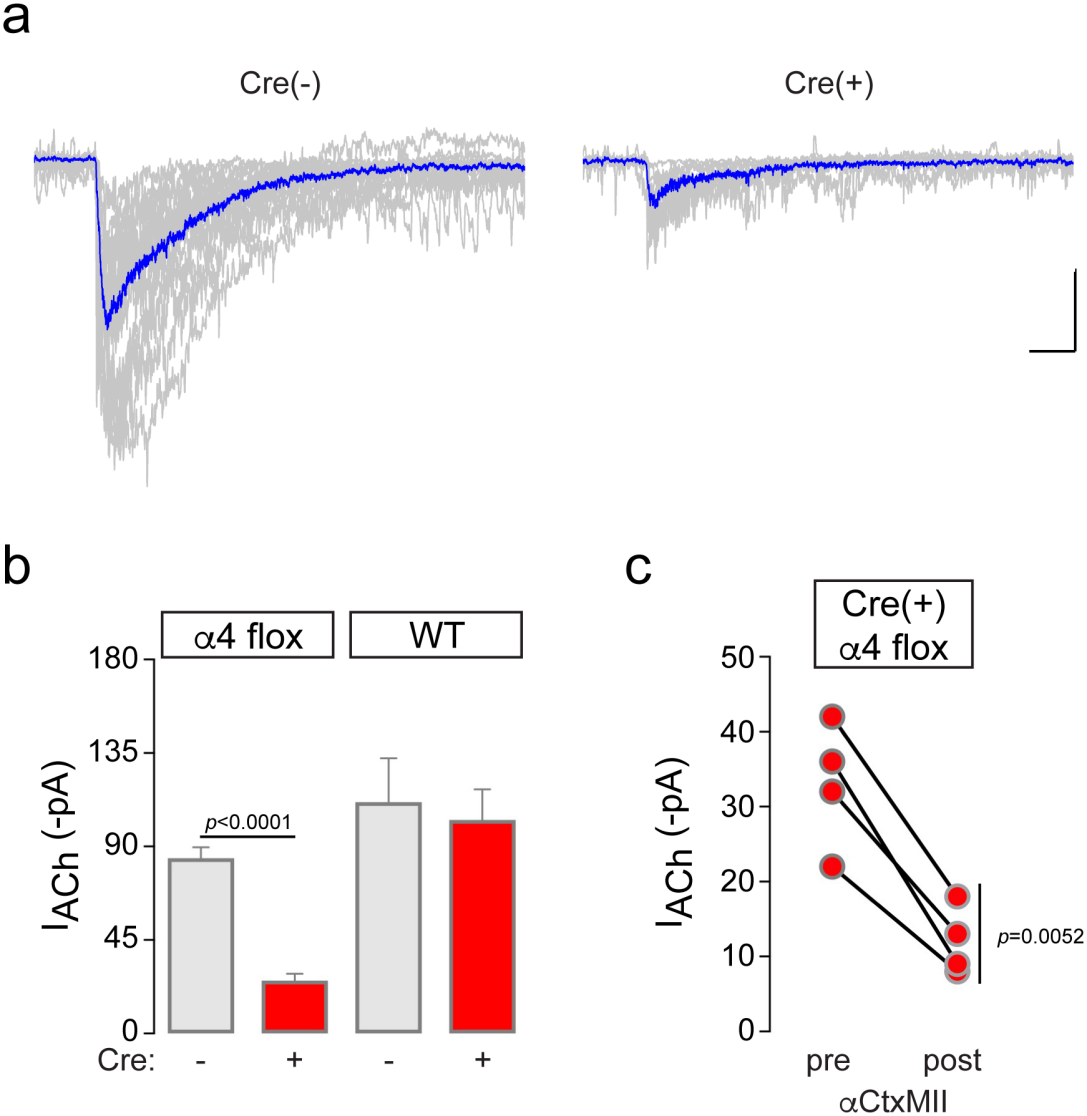
Functional deletion of α4 subunits in VTA. a) Recordings were made from α4-flox;Cre(-) and Cre(+) VTA DA neurons. ACh (1 mM) was applied by pressure ejection and inward currents were recorded. All recorded traces are shown in gray, and an averaged trace is shown in blue. Scale bar: 40 pA, 400 ms. b) Quantification of ACh-evoked currents in AAV-infected mice. Mean inward ACh-evoked currents from α4-flox;Cre(-) (n = 19 cells from n = 9 animals), α4-flox;Cre(+) (n = 14 cells from n = 5 animals), WT;Cre(-) (n = 7 cells from n = 2 animals), and WT;Cre(+) (n = 8 cells from n = 2 animals) cells ± SEM. ****p<0.0001 (unpaired *t*-test, *t* = 8.788). c) Residual ACh-evoked currents in α4-flox;Cre(+) neurons are αCtxMII-sensitive. For Cre(+) responses in (B), αCtxMII (100 nM) was applied by superfusion and ACh was re-applied after 12–15 min. Before-after responses for n = 4 αCtxMII-treated neurons (n = 2 animals) are shown. **p = 0.0052 (paired *t*-test, *t* = 7.348).

To confirm α4* nAChR deletion with a functional assay, we recorded ACh-evoked currents in VTA neurons from α4-flox;Cre(+) mice. First, we measured baseline electrophysiological properties of VTA neurons from α4-flox;Cre(-) and Cre(+) mice. We targeted the lateral VTA, which is reported to contain > 95% DA neurons [40]. To confirm this, we collected cytoplasm via the recording pipette from n = 6 lateral VTA neurons during patch clamp recordings. Following reverse-transcription to produce cDNA from mRNA, we amplified PCR products to determine whether the recorded cell expressed tyrosine hydroxylase (TH) or glutamic acid decarboxylase (GAD65/67). PCR reactions amplifying glyceraldehyde-3-phosphate dehydrogenase (GAPDH) served as an internal control. TH was expressed in 100% (6 of 6) of lateral VTA neurons (S1 Fig), similar to our previous work [28]. No GAD65/67 signal was detected in TH(+) neurons. Based on these results, we conclude that our electrophysiological recordings in lateral VTA (described below) are from DA neurons. There was no difference in resting membrane potential (α4-flox Cre(-): -44.2 ± 1 mV, n = 20; α4-flox Cre(+): -45.4 ± 1 mV, n = 12) or input resistance (α4-flox Cre(-): 747 ± 53 MΩ, n = 17; α4-flox Cre(+): 763 ± 90 MΩ, n = 12) in α4-flox;Cre(+) versus neighboring Cre(-) neurons. Functional nAChR responses were recorded by pressure ejection application of a saturating ACh concentration (1 mM) to voltage clamped neurons. Cre expression resulted in a significant reduction (*p*<0.0001, *t* = 8.788; unpaired *t*-test) in ACh-evoked current amplitude (Fig 4a and 4b). Cre recombinase expression did not alter ACh-evoked currents in control WT mice (*p* = 0.74, *t* = 0.3395; unpaired *t*-test) (Fig 4b). Residual ACh-evoked currents in Cre(+) VTA neurons from α4-flox mice were further inhibited by superfusion of αCtxMII (100 nM), an antagonist of α6β2* nAChRs (*p* = 0.0052, *t* = 7.348; paired *t*-test) (Fig 4c). To confirm that these results were specific to nAChR antagonism and not to receptor run-down or desensitization, we recorded ACh-evoked currents from several Cre(+) VTA neurons before and after a mock antagonist (aCSF without antagonist) superfusion. Responses were not affected by this treatment (*p* = 0.58, *t* = 0.6194; paired *t*-test) (S6 Fig). These results confirm that α6(non-α4)* nAChRs are present on the surface of VTA neurons in α4-flox;Cre(+) mice. All ACh-evoked current results are included in S3 File.

**Fig 5:**
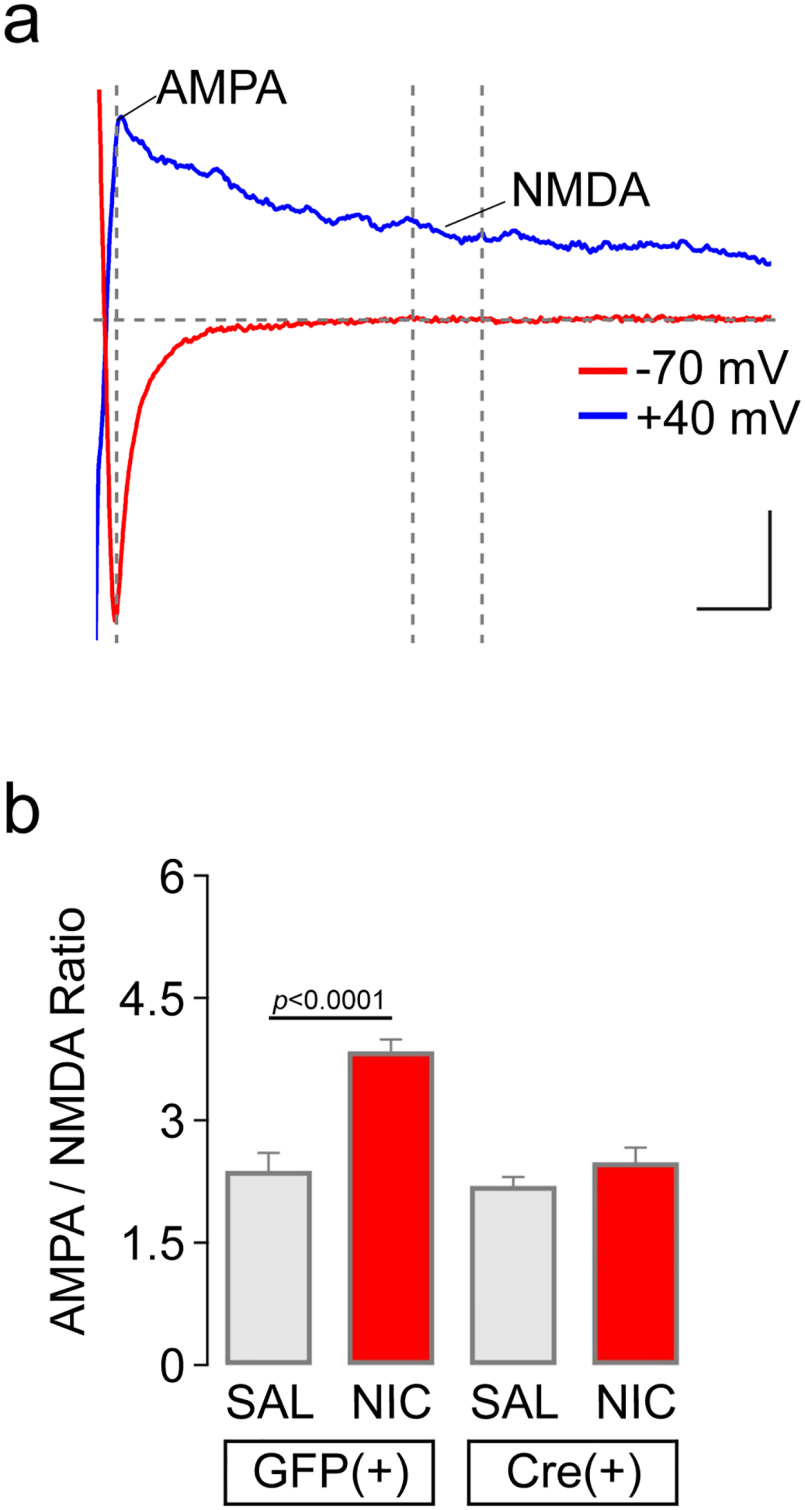
Reduced AMPA/NMDA ratios in α4-flox;Cre(+) VTA DA neurons. a) AMPA/NMDA ratios were measured as described in Materials and Methods. A representative evoked synaptic current in a neuron voltage clamped at -70 mV and +40 mV is shown. b) Quantification of AMPA/NMDA ratios. In each VTA DA neuron from α4-flox;Cre(+) and GFP(+) mice injected with saline (SAL) or nicotine (NIC; 0.17 mg/kg), mean (± SEM) AMPA/NMDA ratio is shown. Replicate cells and number of animals: GFP(+) SAL, n = 8 cells, n = 3 animals; GFP(+) NIC, n = 16 cells, n = 5 animals; Cre(+) SAL, n = 8 cells, n = 3 animals; Cre(+) NIC, n = 7 cells, n = 2 animals. ****p<0.0001 (Bonferroni’s multiple comparison test following two-way ANOVA).

We further tested the idea that a reduction in nAChR sensitivity is a factor contributing to the behavioral phenotypes we report. Brief exposure to nicotine or other drugs of abuse leads to transient synaptic potentiation of glutamate inputs to VTA neurons, which typically manifests itself as an increase in the ratio of AMPA to NMDA receptor currents [41]. We [12] and others [42] have previously shown that a single i.p. injection of nicotine leads to such potentiation, so we tested this effect of nicotine in α4-flox Cre(+) mice. Groups of α4-flox;GFP(+) and α4-flox;Cre(+) mice were handled for several days followed by a single i.p. injection of nicotine (0.17 mg/kg) or saline. One hour after injection, brain slices were prepared and AMPA/NMDA ratios were measured in VTA DA neurons (Fig 5a). Whereas nicotine injection clearly increased the AMPA/NMDA ratio in α4-flox;GFP(+) mice, this effect was blocked in α4-flox;Cre(+) mice (Fig 5b). ANOVA of drug treatment (nicotine dose) (2) x genotype (2) indicated a significant main effect of genotype (*F*(1,35) = 18.05, *p* = 0.0002), a significant main effect of drug treatment (*F*(1,35) = 23.2, *p*<0.0001), and a significant interaction between the two (*F*(1,35) = 10.44, *p* = 0.0027). Bonferroni post-hoc testing revealed a significant increase in AMPA/NMDA ratio in GFP(+) slices from mice injected with nicotine compared to saline (*p*<0.0001). In contrast, there was no difference in AMPA/NMDA ratio in Cre(+) slices from mice injected with nicotine compared to saline (*p*>0.99). All AMPA/NMDA ratio results are included in S4 File.

**Fig 6:**
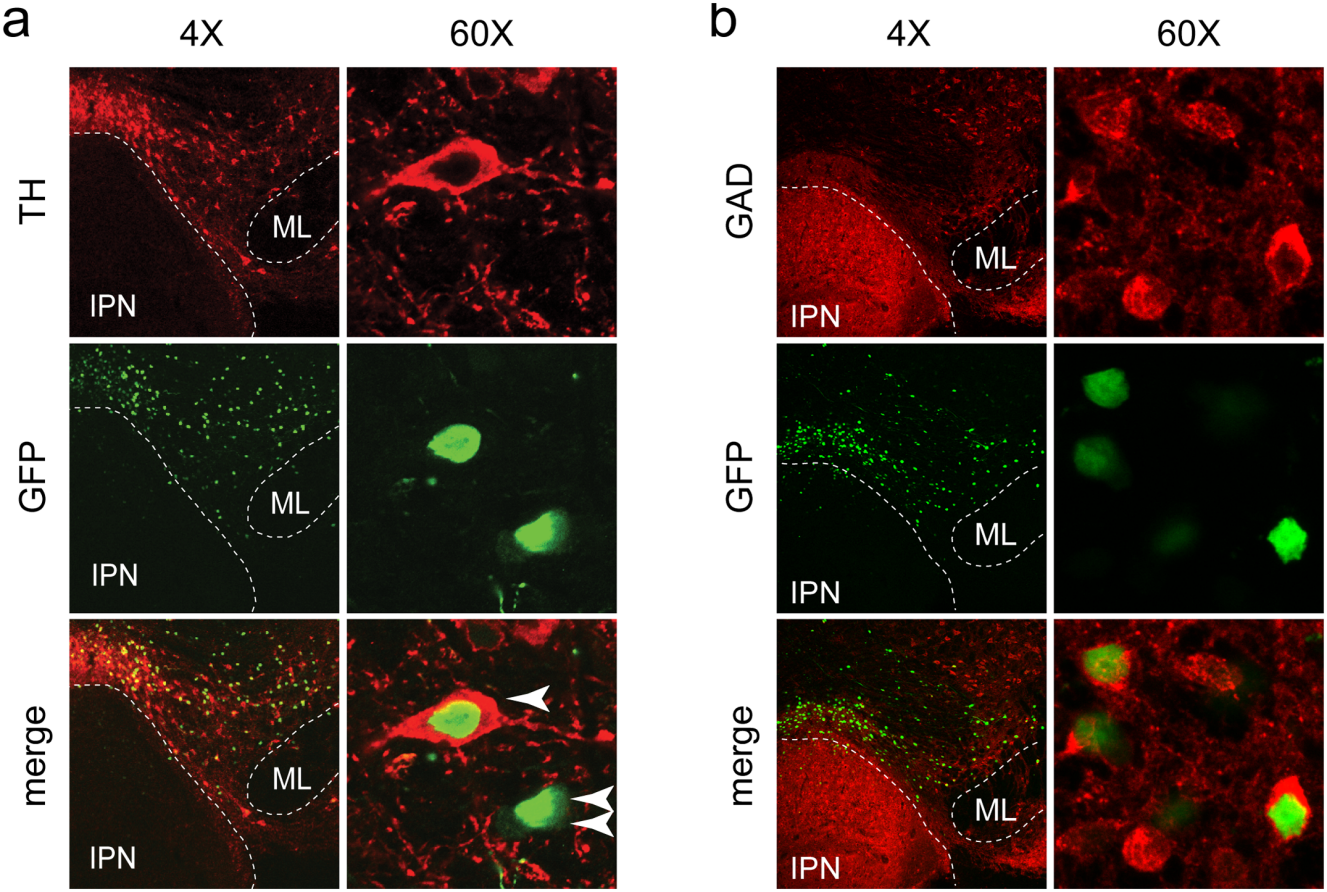
Cre virus infection of VTA DA and GABA neurons. a) Targeting the VTA with AAV vectors. Mice were infused into the VTA with AAV-Cre-GFP vectors. After 2–3 weeks, mice were perfused and brains were sectioned for immunohistochemistry. Anti-GFP staining identifies infected neurons, while anti-TH staining identifies DA neurons. High magnification (60X) images show Cre-GFP expression in TH(+) and TH(-) neurons in VTA, indicated by one or two arrows, respectively. IPN: interpeduncular nucleus, ML: medial lemniscus. b) Cre recombinase expression in VTA GABA neurons. Slices from the infection shown in (a) were stained with anti-GAD65/67 antibodies. Native GFP signal was imaged without anti-GFP staining.

Though previously thought to be composed mainly of dopaminergic neurons, it is now appreciated that the VTA contains neurons producing several different neurotransmitters, including some that co-transmit multiple transmitters [43, 44]. Previous work demonstrated that α4 subunits are expressed in both DA and GABA neurons within the VTA [7], so we determined whether our viral technique resulted in infection of both of these cell types. Indeed, we found robust expression of Cre in tyrosine hydroxylase (TH) immunopositive DA neurons (Fig 6a). Infected TH(-) neurons were also identified, suggesting Cre expression in VTA GABA neurons. Anti-GAD staining confirmed this (Fig 6b). These staining experiments suggest that our behavioral phenotypes are a result of combined disruption of α4* nAChR function in VTA DA and GABA neurons.

## Discussion

Nicotinic ACh receptors in ventral midbrain DA circuits are complex in their localization and composition, and we addressed the shortcomings in our understanding of this system by selectively eliminating vMB α4 nAChR subunits with a conditional knockout approach that bypassed potential developmental artifacts in nAChR subunit expression. The main contributions of this study are results obtained by juxtaposition of nicotine oral SA, nicotine CPP, and targeted electrophysiology in mice with a discrete nAChR loss-of-function mutation.

Interestingly, Cre(+) mice showed greater nicotine intake (significant at the highest concentration offered) but did not demonstrate nicotine CPP at the selected nicotine dose of 0.25 mg/kg. The assays differ with respect to controllability of drug exposure, route of drug administration, kinetics of nicotine exposure, number of nicotine exposures, and variability of nicotine dosage [45]. Oral self-administration assays are frequently used to assess the motivational effects of abused drugs and have the advantage of allowing the animal to control their own intake of the drug, thereby providing a relatively direct index of its reinforcing value. However, because this assay involves consummatory behavior, many other factors, such as taste, may interfere with the ability to achieve pharmacologically relevant blood levels of drug. In contrast, the place conditioning assay avoids the oral route of administration by relying on Pavlovian conditioning to produce conditioned approach or avoidance responses to environmental cues previously associated with a drug’s pharmacological effects. A primary advantage of this procedure is that it allows precise control over drug dose exposure, and, when used in conjunction with oral SA procedures, can help elucidate whether differences in oral drug intake are influenced by centrally-mediated neurobiological mechanisms that control sensitivity to a drug’s motivational (rewarding and/or aversive) effects [46]. It should be noted that both CPP and oral SA mice were single housed, which reduces the possibility that social isolation stress differentially influenced one data set over the other. Also, mice were food restricted in the CPP assay but not in the oral SA assay.

Nicotine oral SA is a widely-used procedure in rodents. Nicotine is maintained in the plasma at concentrations analogous to human smokers when ingested by rodents [47], and modest doses are sufficient to cause nAChR upregulation [48], a hallmark of nicotine dependence [49, 50]. During free-access studies with increasing concentrations of nicotine presented in the home cage every few days, mice will titrate their consumption in a presumptive attempt to maintain nicotine levels in an optimal, reinforcing range [18]. In a fading study, mice compensated for declining nicotine concentrations by increasing their consumption [47]. Nicotine exposure in drinking water can lead to tolerance [48], another key feature of nicotine dependence. Importantly, rats will press a lever to gain access to oral nicotine solutions [51, 52]. Furthermore, systemic mecamylamine pre-treatment *increased* nicotine operant oral SA, which likely reflected a compensatory behavioral response to mecamylamine’s pharmacological blockade of nAChRs involved in nicotine’s reinforcing effects [52]. Interestingly, mecamylamine also enhanced preference for cigarettes in human smokers [53–55]. These results, coupled with the robust and repeatable self-titration of nicotine that occurs in nicotine oral SA [18] and intravenous SA [25, 56], are helpful in interpreting our oral SA results. α4-flox mice appeared to regulate their nicotine intake as nicotine concentration increased (Fig 2). The enhanced nicotine intake in Cre(+) mice (statistically significant at the highest nicotine concentration of 200 μg/mL) could reflect an attempt to compensate for reduced sensitivity to nicotine’s rewarding effects in vMB reward circuits. Reduced sensitivity was evident in our nicotine CPP results and our electrophysiological analyses demonstrating nAChR loss-of-function (Figs 4 and 5). Alternatively, enhanced nicotine intake at 200 μg/mL could reflect reduced sensitivity to the aversive effects of nicotine. Future work designed to dissect the aversive aspect of nicotine exposure will be required to uncover the mechanism behind increased nicotine intake in Cre(+) mice.

Prior work strongly implicates α4* nAChRs in the acquisition of nicotine CPP. Nicotine CPP is attenuated in both α4 global KO mice [57] as well as conditional KO mice lacking expression of α4 specifically in dopamine transporter (DAT)-expressing neurons [16]. Using a complementary approach involving mice expressing hypersensitive α4* nAChRs, Lester and colleagues demonstrated that selective activation of these receptors is sufficient for acquisition of nicotine CPP [1]. Re-expression of α4 subunits in the VTA of α4 KO mice restores nicotine self-administration and nicotine-elicited alterations in DA neuron activity [2, 3]. Ours is the first study to report that α4 removal from the vMB of adult mice blocks nicotine CPP, which is consistent with these prior studies.

Our electrophysiological analysis of nAChR function in Cre(-) and Cre(+) VTA DA neurons point to α4β2* nAChRs as the dominant nAChR in somatodendritic areas of these cells. Although consistent with previous results [58, 59], the ~75% reduction in ACh-evoked current amplitude that we measured in α4-flox;Cre(+) VTA cells (Fig 4a and 4b) represents a substantial reduction in surface receptors. α6β2* nAChRs account for a majority of the residual nAChRs in these cells (Fig 4c) [58, 60], suggesting only a slight additional contribution by subtypes such as α7 or β4* nAChRs that have previously been suggested to reside in VTA neurons [61, 62]. Though we recorded in an area (lateral VTA) that is enriched in tyrosine hydroxylase expressing (DA) neurons (S1 Fig), our AAV approach also targeted α4 for deletion in VTA GABA neurons (Fig 6). These cells are strongly modulated by cholinergic tone in part through α4* nAChR activity [7, 63], and their activity potently modulates nearby DA neurons through local VTA microcircuits [64]. Further, afferent inputs to, and efferent connections from VTA GABA neurons are distinct from other VTA cell types [65, 66]. Viral approaches that manipulate nAChR expression in neurotransmitter-defined VTA cell types of adult animals, which are beginning to be employed [20], will be valuable for understanding cholinergic regulation of the reward system.

α6* subunits are also found in a large subset of vMB nAChRs [58, 67] and modulate DA release [14, 68], locomotor behavior [9, 12–14], nicotine CPP [57], nicotine intravenous SA [2], and intracranial SA of nicotine [3]. It is important to note that in our system, α6β2*(non-α4) nAChRs are left intact, allowing us to draw conclusions about the sufficiency of these receptors in the behavioral assays we conducted. Consequently, we conclude that activation of α6β2*(non-α4) nAChRs, a relatively low-sensitivity nAChR [69], is insufficient to support acquisition of CPP (at 0.25 mg/kg) when α4 subunits are absent. Also, we cannot rule out the possibility that it is the activity of α6β2*(non-α4), rather than the lack of α4* nAChR activity, that controls elevated nicotine oral SA in α4-flox;Cre(+) mice.

## Conclusions

We report that selective deletion of α4 nAChR subunits in vMB neurons of adult mice results in reduced sensitivity to nicotine in behavioral and electrophysiological experiments. This loss-of-function renders systemic injections of 0.25 mg/kg nicotine ineffective during Pavlovian conditioning, perhaps due to a lack of transient synaptic potentiation that is known to be crucial for early responses to drugs of abuse [41, 70]. Ventral midbrain α4 loss-of-function also enhances intake of high nicotine concentrations. Since drugs are actively being developed to antagonize vMB nAChRs as smoking cessation therapeutics, and since we have identified a circumstance that leads to increased nicotine intake instead of the desired decrease, future studies should also work to identify the specific nAChR subtypes involved in regulating nicotine self-administration in defined midbrain circuits.

## Supporting information

S1 Fig Verification of DA neurons in lateral VTA. For a subset (n = 6) of lateral VTA neurons, cytoplasm was captured, RNA was prepared, and cDNA was synthesized via reverse transcription. Primer pairs for amplification of GAD65/67 (GABA neuron marker), tyrosine hydroxylase (TH), and a housekeeping gene (GAPDH) were used in PCR reactions using cDNA as a template. A representative gel image (6 of 6 neurons showed similar results) is shown for a lateral VTA neuron, and a negative control sample confirms the specificity of the result.
(TIF)

S2 Fig Related to Fig 2. Male oral SA data. Data shown in Fig 2 were disaggregated by sex. Mean values for each of the following measures are shown at each nicotine concentration in male GFP(+) and Cre(+) α4flox mice: (a) nicotine intake mass (mg/kg/24 h); (b) nicotine preference ratio; (c) nicotine intake volume (mL/kg/24 h); (d) water intake volume (mL/kg/24 h); (e) total fluid intake volume (mL/kg/24 h); (f) body weight (g).
(TIF)

S3 Fig Related to Fig 2. Female oral SA data. Data shown in Fig 2 were disaggregated by sex. Mean values for each of the following measures are shown at each nicotine concentration in female GFP(+) and Cre(+) α4flox mice: (a) nicotine intake mass (mg/kg/24 h); (b) nicotine preference ratio; (c) nicotine intake volume (mL/kg/24 h); (d) water intake volume (mL/kg/24 h); (e) total fluid intake volume (mL/kg/24 h); (f) body weight (g).
(TIF)

S4 Fig Related to Fig 2. Nicotine intake scatter plots, disaggregated by sex. Data shown in S2a Fig (a) and S3a Fig (b) was re-plotted as a scatter plot.
(TIF)

S5 Fig Related to Fig 3. CPP data disaggregated by sex and virus type. Total time spent by each mouse in the nicotine-paired chamber is shown for the pre-test and post-test days using before-after scatter plots.
(TIF)

S6 Fig Related to Fig 4. Run-down / desensitization control. ACh (1 mM)-evoked currents were measured in Cre(+) VTA neurons from α4-flox;Cre(+) mice before and after mock antagonist application to probe for any non-specific run-down of inward currents. (a) Representative traces from one cell stimulating with 1 mM ACh showing a control response (black trace) and a response following 14 min superfusion of drug vehicle aCSF. Scale bar: 3 s, 15 pA (b) A before-after plot is shown for responses from n = 4 cells from n = 3 animals. A paired t-test revealed no significant effect of aCSF treatment (*t* = 0.6194, *p* = 0.58).
(TIF)

S1 File DRENAN Oral SA.xlsx. Complete data set for results reported in Fig 2, S2, S3 and S4 Figs.
(XLSX)

S2 File DRENAN CPP.xlsx. Complete data set for results reported in Fig 3b–3e.
(XLSX)

S3 File DRENAN ACh currents.xlsx. Complete data set for results reported in Fig 4b and 4c and S6 Fig.
(XLSX)

S4 File DRENAN AMPA NMDA.xlsx. Complete data set for results reported in Fig 5b.
(XLSX)
