# Supplementary figures and images for: Altered nicotine reward-associated behavior following α4 nAChR subunit deletion in ventral midbrain
Source: PLoS One. 2017 Jul 31;12(7):e0182142. doi: 10.1371/journal.pone.0182142 (PMC5536316; doi:10.1371/journal.pone.0182142)

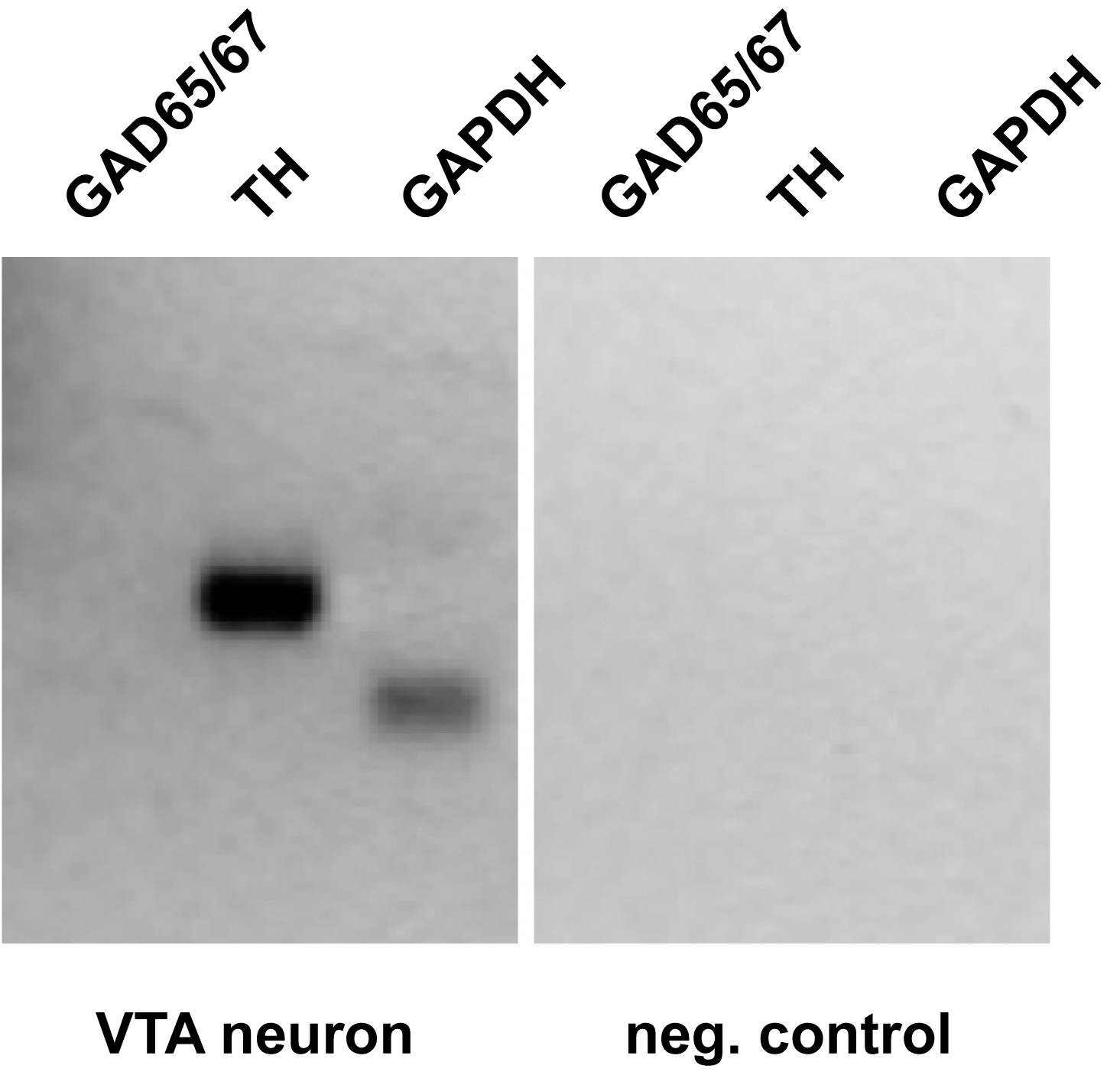

Supplement: S1 Fig — For a subset (n = 6) of lateral VTA neurons, cytoplasm was captured, RNA was prepared, and cDNA was synthesized via reverse transcription. Primer pairs for amplification of GAD65/67 (GABA neuron marker), tyrosine hydroxylase (TH), and a housekeeping gene (GAPDH) were used in PCR reactions using cDNA as a template. A representative gel image (6 of 6 neurons showed similar results) is shown for a lateral VTA neuron, and a negative control sample confirms the specificity of the result. (TIF) [file pone.0182142.s001.tif]

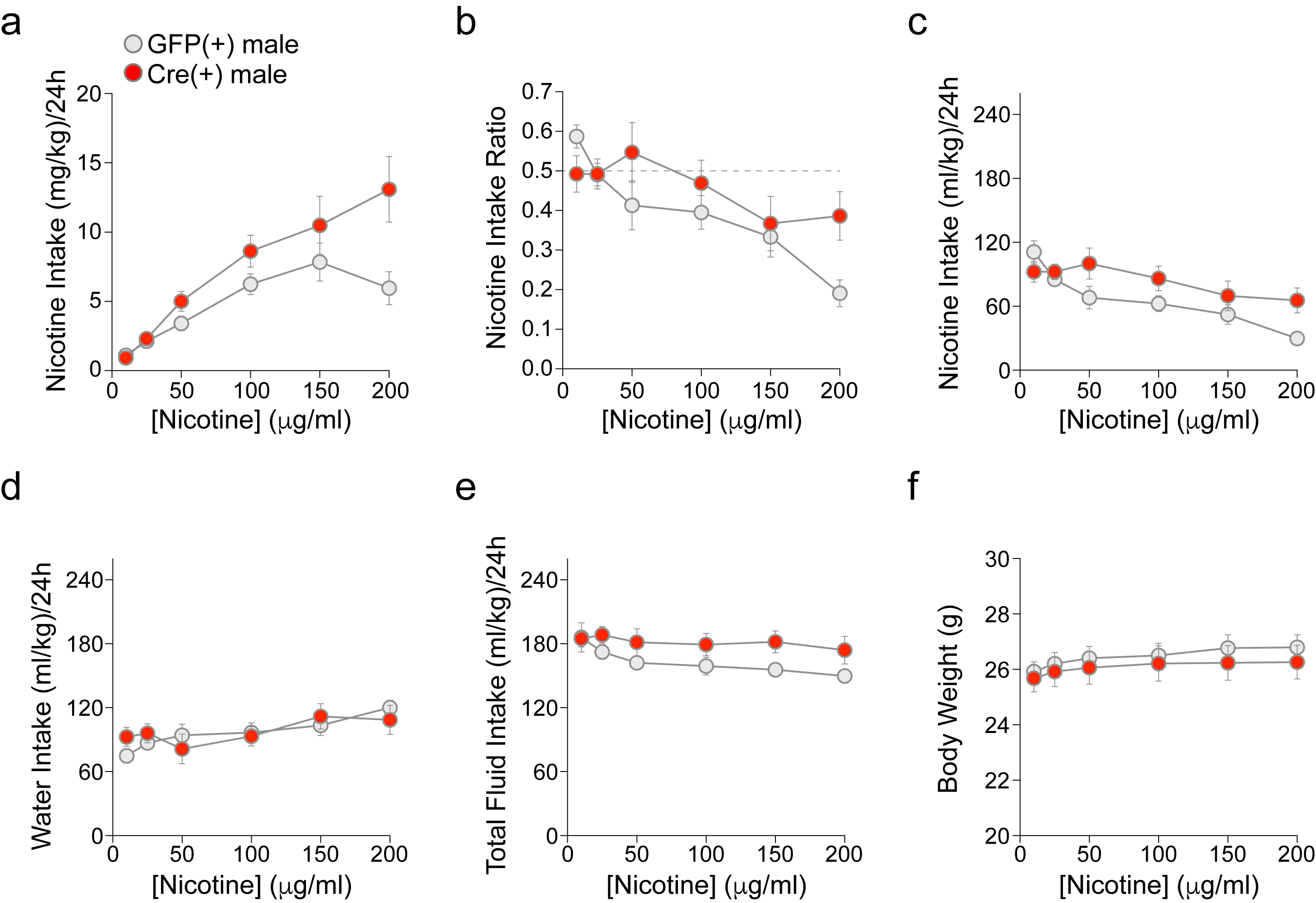

Supplement: S2 Fig — Data shown in Fig 2 were disaggregated by sex. Mean values for each of the following measures are shown at each nicotine concentration in male GFP(+) and Cre(+) α4flox mice: (a) nicotine intake mass (mg/kg/24 h); (b) nicotine preference ratio; (c) nicotine intake volume (mL/kg/24 h); (d) water intake volume (mL/kg/24 h); (e) total fluid intake volume (mL/kg/24 h); (f) body weight (g). (TIF) [file pone.0182142.s002.tif]

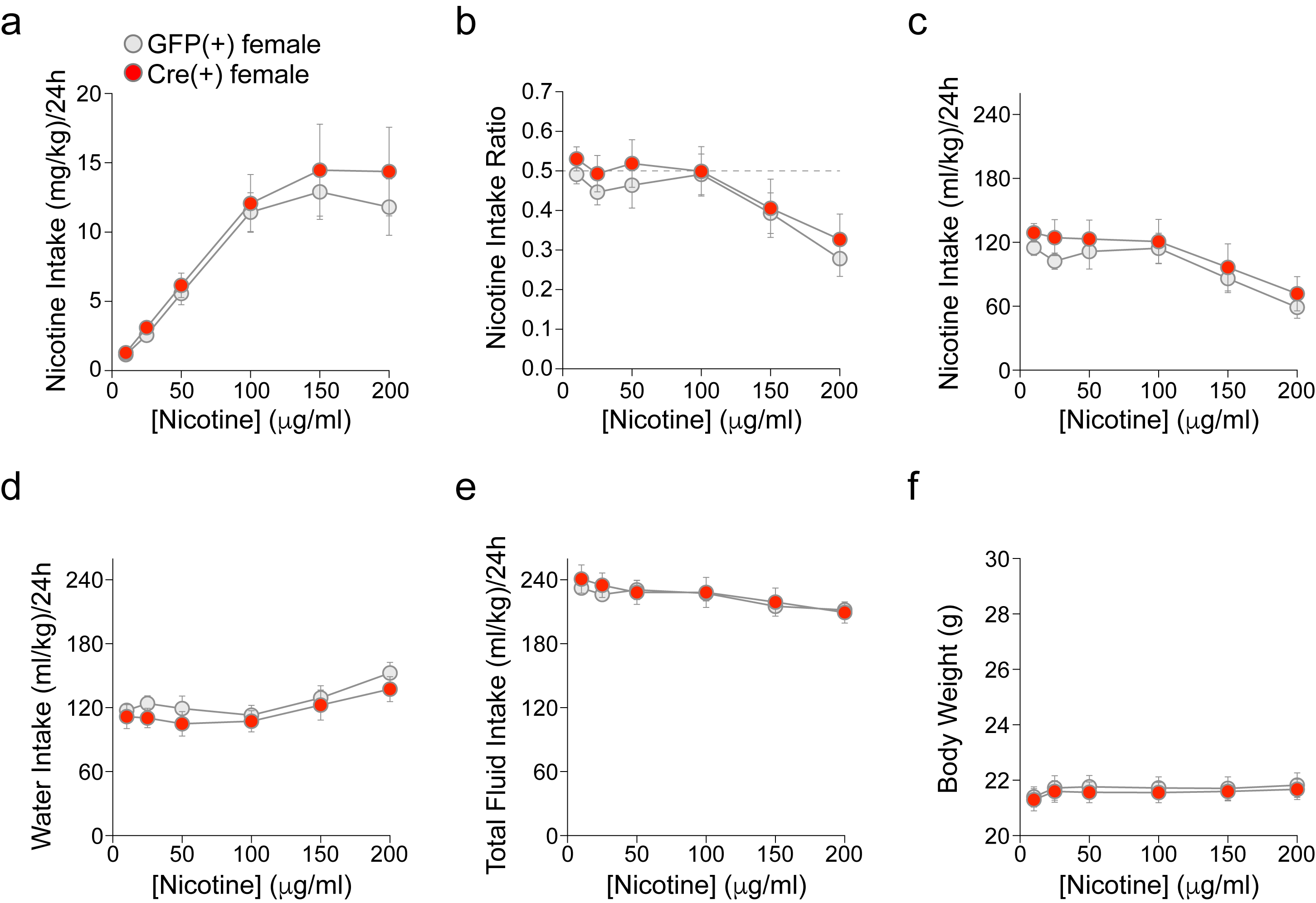

Supplement: S3 Fig — Data shown in Fig 2 were disaggregated by sex. Mean values for each of the following measures are shown at each nicotine concentration in female GFP(+) and Cre(+) α4flox mice: (a) nicotine intake mass (mg/kg/24 h); (b) nicotine preference ratio; (c) nicotine intake volume (mL/kg/24 h); (d) water intake volume (mL/kg/24 h); (e) total fluid intake volume (mL/kg/24 h); (f) body weight (g). (TIF) [file pone.0182142.s003.tif]

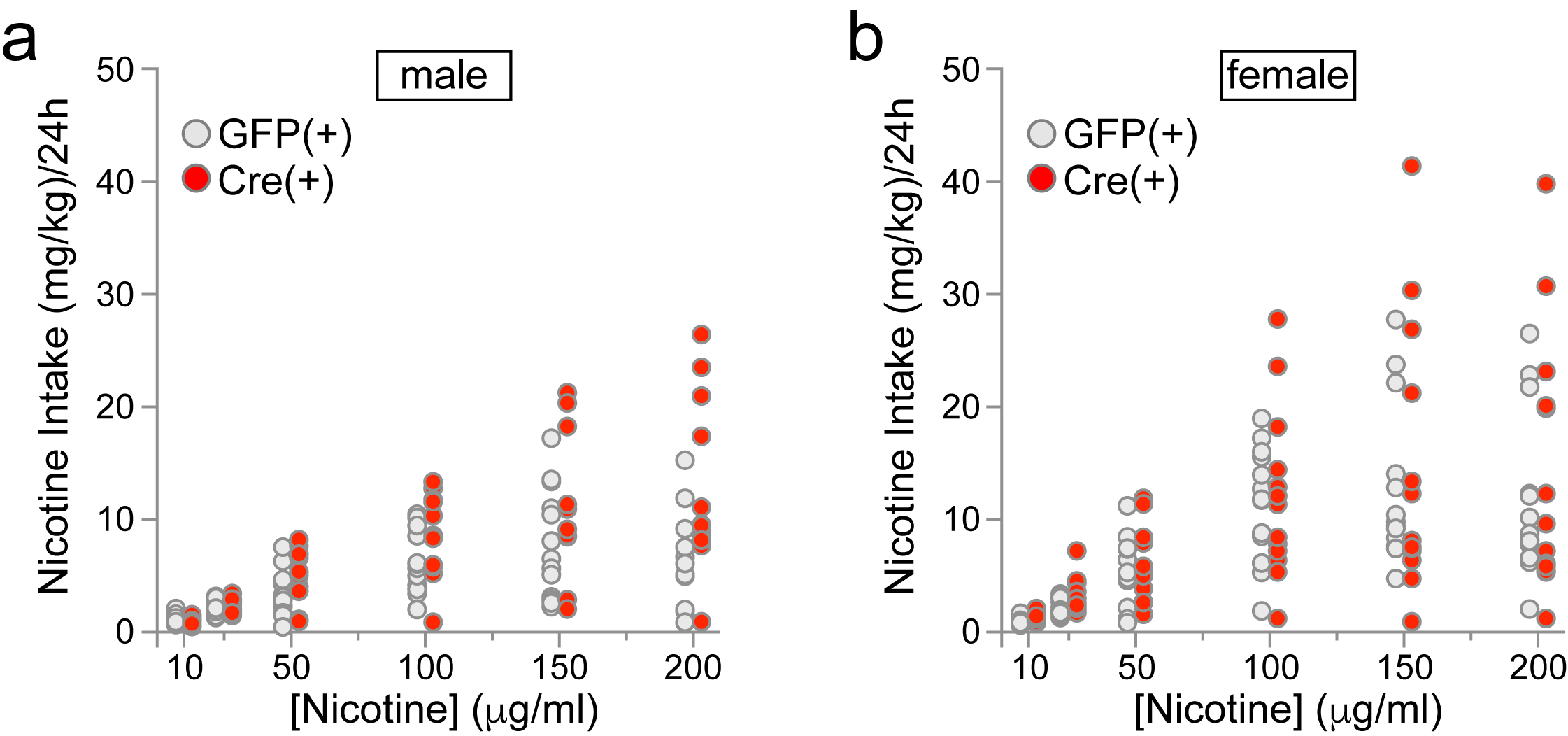

Supplement: S4 Fig — Data shown in S2a Fig (a) and S3a Fig (b) was re-plotted as a scatter plot. (TIF) [file pone.0182142.s004.tif]

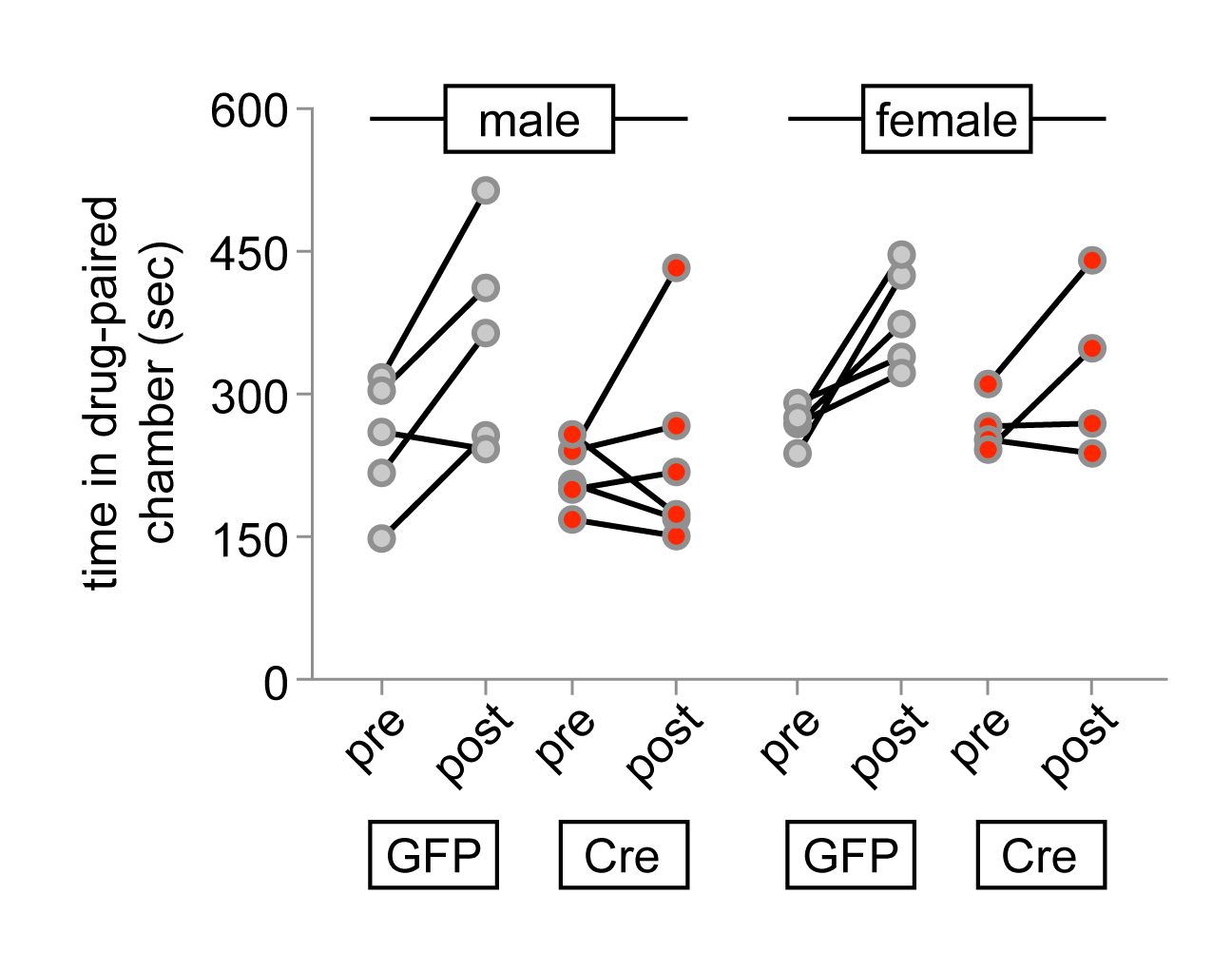

Supplement: S5 Fig — Total time spent by each mouse in the nicotine-paired chamber is shown for the pre-test and post-test days using before-after scatter plots. (TIF) [file pone.0182142.s005.tif]

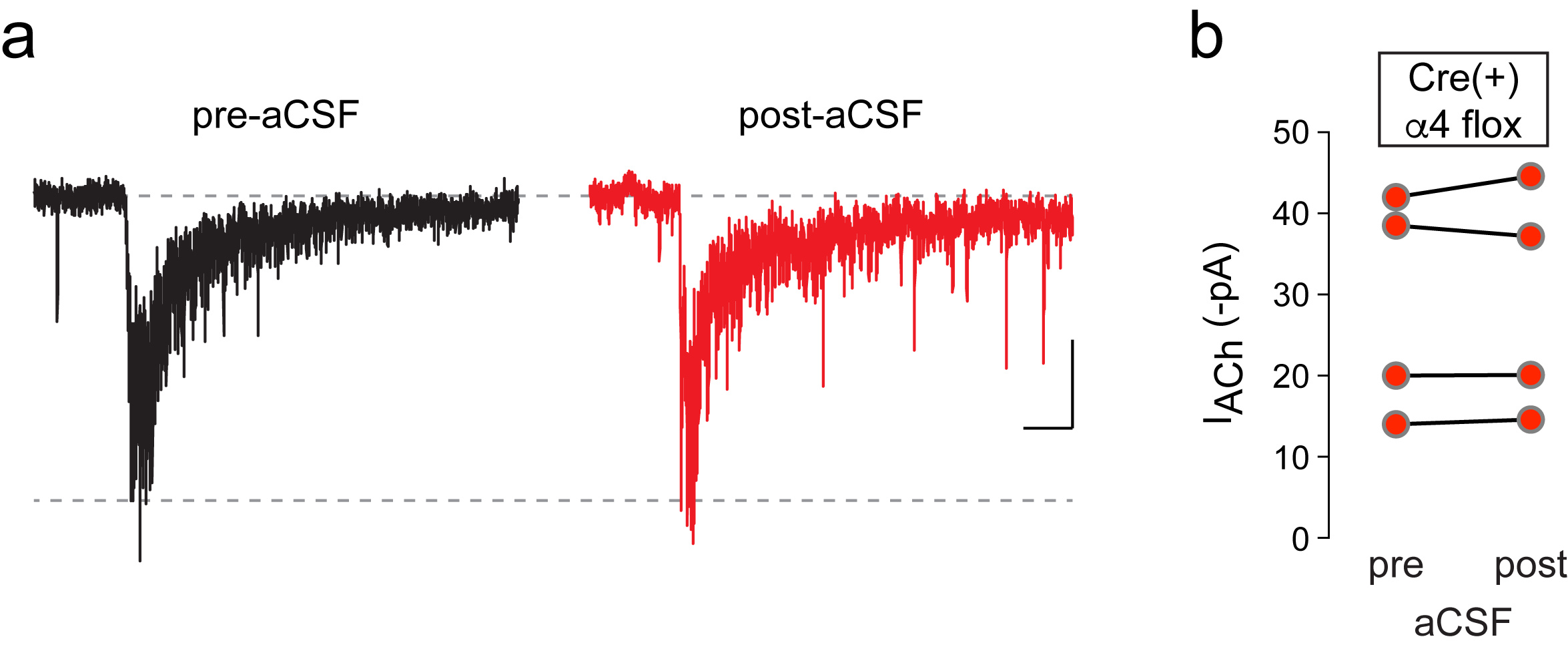

Supplement: S6 Fig — ACh (1 mM)-evoked currents were measured in Cre(+) VTA neurons from α4-flox;Cre(+) mice before and after mock antagonist application to probe for any non-specific run-down of inward currents. (a) Representative traces from one cell stimulating with 1 mM ACh showing a control response (black trace) and a response following 14 min superfusion of drug vehicle aCSF. Scale bar: 3 s, 15 pA (b) A before-after plot is shown for responses from n = 4 cells from n = 3 animals. A paired t-test revealed no significant effect of aCSF treatment (t = 0.6194, p = 0.58). (TIF) [file pone.0182142.s006.tif]
